# Intracellular accumulation capacity of gadoxetate: initial results for a novel biomarker of liver function

**DOI:** 10.1038/s41598-020-75145-y

**Published:** 2020-10-22

**Authors:** Ute Lina Fahlenkamp, Katharina Ziegeler, Lisa Christine Adams, Sarah Maria Böker, Günther Engel, Marcus Richard Makowski

**Affiliations:** 1 Department of Radiology, Charité-Universitätsmedizin Berlin, Charitéplatz 1, 10117 Berlin, Germany; 2 Department of Radiology, Klinikum rechts der Isar der TU München, Ismaninger Straße 22, 81675 Munich, Germany

**Keywords:** Biomarkers, Gastroenterology, Molecular medicine

## Abstract

Previous studies have shown gadoxetate disodium’s potential to represent liver function by its retention in the hepatobiliary phase. Additionally, in cardiac imaging, quantitative characterization of altered parenchyma is established by extracellular volume (ECV) calculation with extracellular contrast agents. Therefore, the purpose of our study was to evaluate whether intracellular accumulation capacity (IAC) of gadoxetate disodium derived from ECV calculation provides added scientific value in terms of liver function compared to the established parameter reduction rate (RR). After local review board approval, 105 patients undergoing standard MR examination with gadoxetate disodium were included. Modified Look-Locker sequences were obtained before and 20 min after contrast agent administration. RR and IAC were calculated and correlated with serum albumin, as a marker of synthetic liver function. Correlation was higher between IAC and albumin, than between RR and albumin. Additionally, capacity of both RR and IAC to distinguish between patients with or without liver cirrhosis was investigated, and differed significantly in their respective means between patients with cirrhosis and those without. We concluded, that the formula to calculate ECV can be transferred to calculate IAC of gadoxetate disodium in hepatocytes, and, thereby, IAC may possibly qualify as an imaging-based parameter to estimate synthetic liver function.

## Introduction

For more than 20 years it has been established, that magnetic resonance imaging (MRI) with gadoxetate disodium has the potential to evaluate hepatic function^1–3^. While initially, calculations were based on signal intensities, later on, mapping techniques, which are less dependent on the type of the scanner or the sequence and well known from cardiac imaging, were also introduced in liver imaging^4–6^. Based on accordingly acquired relaxation times, a reduction rate (RR) was determined, which describes the ratio between enhancement of liver parenchyma after application of gadoxetate disodium and native liver parenchyma^4,7^. Still, the reduction rate does not account for the effect blood may have on relaxation time, which may vary due to factors such as the amount of gadolinium contrast injected, post contrast scan times, renal function, hematocrit, and B_o_ field. Normalizing hepatic T1 in relationship to blood T1 may diminish much of these complexities^8–10^.

Cardiac MR imaging has shown the potential of myocardial extracellular volume fraction (ECV) as the percent of tissue composing the extracellular space^9^, and the approach has been translated to hepatic imaging in the experimental field^11^. Due to the property of gadoxetate disodium to be taken up by hepatocytes according to the amount of functioning organic-anion-transport proteins (OATP), in the equilibrium phase, extracellular concentrations are very low, whereas intracellularly, the contrast agent is accumulated. Therefore, for gadoxetate disodium, we hypothesized that the intracellular accumulation capacity (IAC) could be calculated according to the established formula for the extracellular space with a minor correction made due to retained contrast agent within the blood and the extracellular space in the hepatobiliary phase^11^.

The purpose of the present exploratory study was to evaluate whether the well-established method to estimate ECV can be transferred to calculation of IAC using the hepatocellular contrast agent gadoxetate disodium, and, if so, whether this method might have advantages over the calculation of the reduction rate.

## Material and methods

### Study population

This prospective cohort study was approved by and registered with the local ethics committee (Ethikkommission der Charité, Ethikausschuss I am Campus Charité-Mitte, EA1/334/16). From January 2017 to November 2019, patients referred for MR examination of the liver with gadoxetate disodium were evaluated for study participation. Exclusion criteria were age younger than 18 years, pregnancy, metallic implants or functional devices not eligible for MR examination, claustrophobia, a history of allergic reaction to Gd-EOB-DTPA, and a glomerular filtration rate below 30 ml/min. With written informed consent to participate in the study, 105 patients (51 males and 54 females; mean age 57.03 years) were included in the study.

All included patients underwent the standard liver MR imaging protocol using the hepatocyte-specific contrast agent gadoxetate disodium (Gd-EOB-DTPA, Primovist), and the study sequences as specified below. All methods were carried out in accordance with the relevant guidelines and regulations.

### Imaging protocol

MR imaging was performed on a 1.5 T scanner (Avanto, Siemens Medical Solutions, Erlangen, Germany) equipped with a 32-channel body-phased-array coil. The standard liver MR imaging protocol using the hepatocyte specific contrast agent gadoxetate disodium comprises an axial T1-weighted spin echo sequence, an axial fat-saturated T2-weighted turbo spin echo sequence acquired with a 2D navigator for abdominal imaging, an axial T1-weighted dual echo sequence, axial T1 VIBE (volume-interpolated breath-hold) sequences for dynamic imaging before and 15 s, 55 s and 2, 5, 10 and 20 min after contrast agent administration and a coronally orientated T1 VIBE sequence for the hepatobiliary phase at least 20 min after contrast agent administration^12^.

### Study sequences

Apart from the clinical routine image protocol, patients received steady-state precession readout single-shot Modified Look-Locker inversion recovery (MOLLI) sequences in the axial plane before contrast agent administration and throughout the hepatobiliary phase.

T1 maps were calculated automatically on a pixel-by-pixel basis, and displayed on a 12-bit lookup table with a visible color-coded map, on which the signal intensity of each of the pixels reflects their absolute T1 value.

The imaging parameters for the study sequence are shown in Table 1.

**Table 1:** Imaging parameters of the study sequence.

Sequence	MOLLI
Scan plane	Axial
Voxel size (mm^3^)	2.4 × 1.6 × 6.0
Number of slices	3
Slice thickness (mm)	6
TR/TE (ms)	912/1.08
Averages	1
FoV (mm)	320
Flip angle (°)	35
Bandwidth (Hz/Px)	1028
Fat saturation	None
Parameter map type	T1 map
Number of inversions	3
MOLLI TI start (ms)	90
MOLLI TI increment (ms)	80
MOLLI trigger delay (ms)	160

*MOLLI* modified Look-Locker Inversion Recovery, *TR* repetition time, *TE* echo time, *FoV* field of view, *TI* inversion time.

### Image analysis

All images were analysed using PACS workstations (Centricity Radiology; GE Healthcare) by a radiologist with 12 years of experience in MR imaging. MOLLI images were assessed by a single reader, blinded to the paraclinical including histopathological findings, if present. Regions of interest of maximal size were placed in the liver parenchyma on native as well as on post-contrast MOLLI images. ROI placement was chosen avoiding larger vessels or focal lesions to avoid partial volume averaging. Additionally, ROIs were placed in the abdominal aorta both on precontrast and postcontrast images.

### Clinical and paraclinical parameters

Patients’ electronic medical records were searched for history of liver disease and documented pertinent laboratory data (hematocrit and albumin, where available).

### Calculation of intracellular accumulation capacity

Calculation of the ECV normalized for hematocrit using an extracellular contrast agent was described elsewhere^9,11,13,14^:

$$ ECV = \frac{{\left( {1/T1 tissue\;post {-} 1/T1\;tissue\;pre} \right)}}{{\left( {1/T1\;blood\;post {-} 1/T1\;blood\;pre} \right) }}*\left( {1 - hematocrit} \right). $$

**Figure 1:**
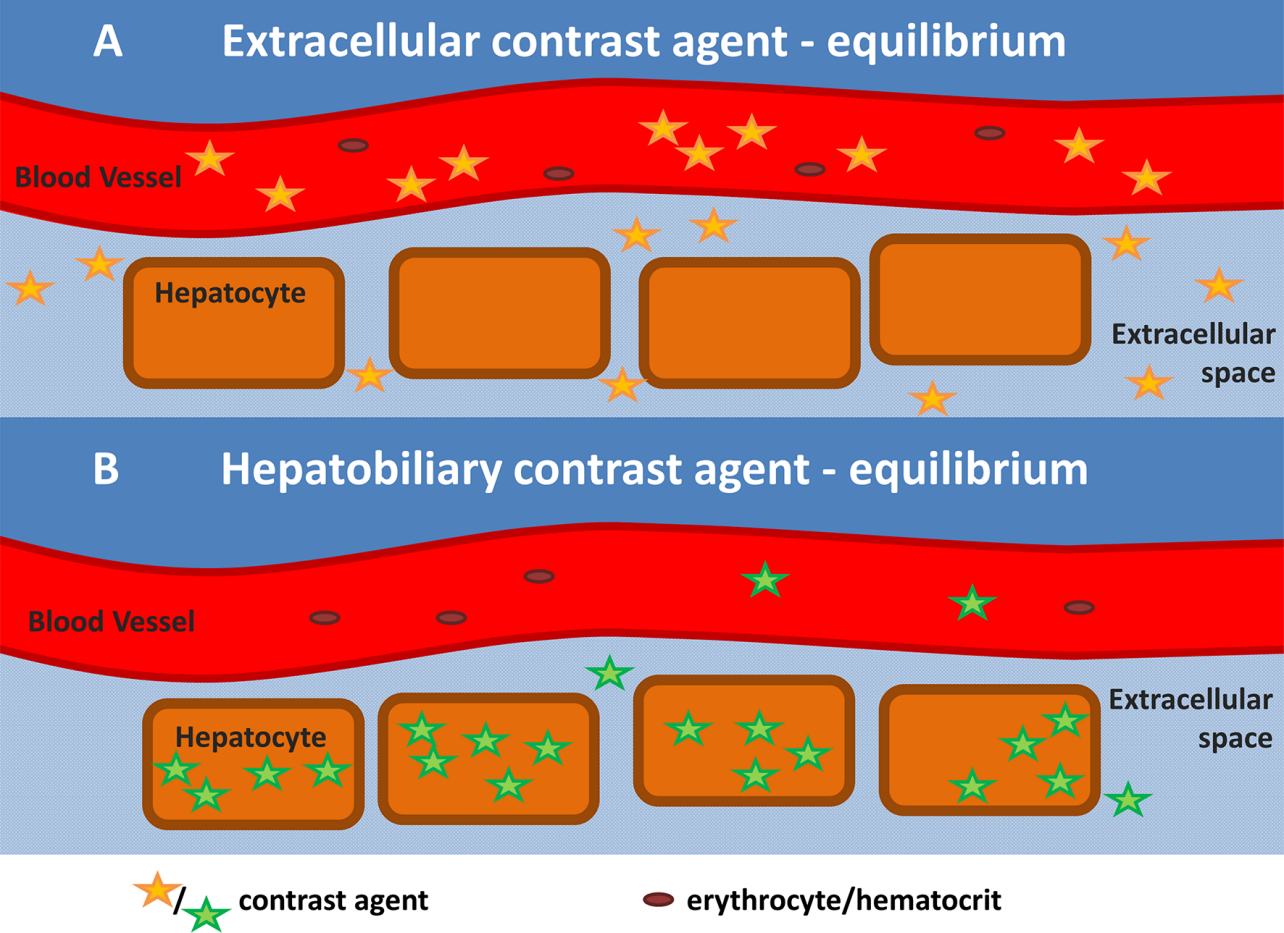
The calculation of ECV implies an equilibrium of gadolinium-based contrast agents between the extracellular interstitium and the intravascular compartment. For IAC, one can assume a low-level equilibrium between intravascular und extracellular compartment, and a mainly intracellular storage.

For hepatocyte specific contrast agents, one may assume a low-level equilibrium between intravascular und extracellular compartment, and a mainly intracellular storage. An illustration can be found in Fig. 1.

Therefore, the above-mentioned formula may allow for calculation of IAC, with a minor correction by subtracting the low-level amount of extracellular contrast agent.

$$ ICV = ECV - {{\left( {1/T1\;blood\;post - 1/T1\;blood\;pre} \right)} \mathord{\left/ {\vphantom {{\left( {1/T1\;blood\;post - 1/T1\;blood\;pre} \right)} {\left( {1 - hematocrit} \right)}}} \right. \kern-\nulldelimiterspace} {\left( {1 - hematocrit} \right)}} $$

The reduction rate was calculated as described elsewhere^4^:

$$ Reduction\;rate\left( \% \right) = \left[ {{\raise0.7ex\hbox{${\left( {T1\;liver\;pre - T1\;liver\;post} \right)}$} \!\mathord{\left/ {\vphantom {{\left( {T1\;liver\;pre - T1\;liver\;post} \right)} {T1\;liver\;pre}}}\right.\kern-\nulldelimiterspace} \!\lower0.7ex\hbox{${T1\;liver\;pre}$}}} \right]*100 $$

### Statistical analysis

All statistical analyses were performed using SPSS Version 24 (IBM Corporation, New York, USA). For a test of normality, the Shapiro–Wilk test was applied. Accordingly, t-test and Mann–Whitney-U were applied. Correlation analyses were performed with Spearman’s Rho and Pearson’s correlation coefficient. Data are expressed as means and standard deviation (SD).

## Results

Hepatic T1 relaxation times varied between 319.67 and 819.00 ms (mean 564.7 ms) for the native sequences, and between 137.67 and 460.00 ms (mean 219.41 ms) for hepatobiliary sequences. The hematocrit varied between 0.238 and 0.492 l/l (mean 564.72 l/l). Albumin values were available in 69 of patients and varied between 20 and 48 g/l (mean 39.89 g/l).

**Figure 2:**
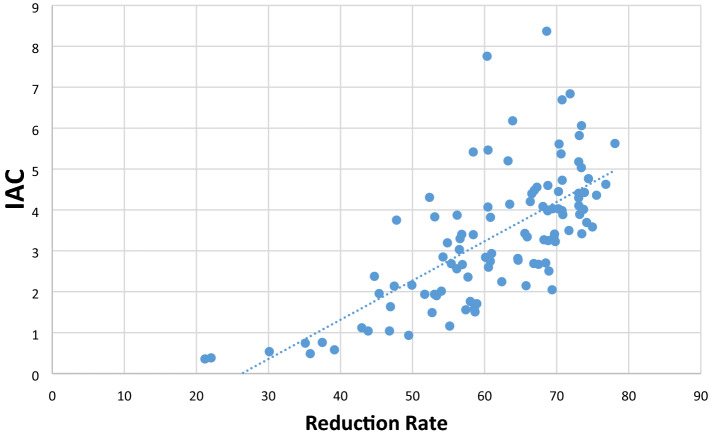
Scatterplot illustrating correlation between IAC and reduction rate.

IAC showed a mean of 3.33 with a minimum of 0.36 and a maximum of 8.37. Reduction rate varied between 21.19 and 78.09 with a mean of 61.06. Correlation between both measures was strong^15^ with a Pearson’s r of 0.697 (p < 0.001)—a visualization of this correlation is given in Fig. 2.

### Cirrhosis

**Figure 3:**
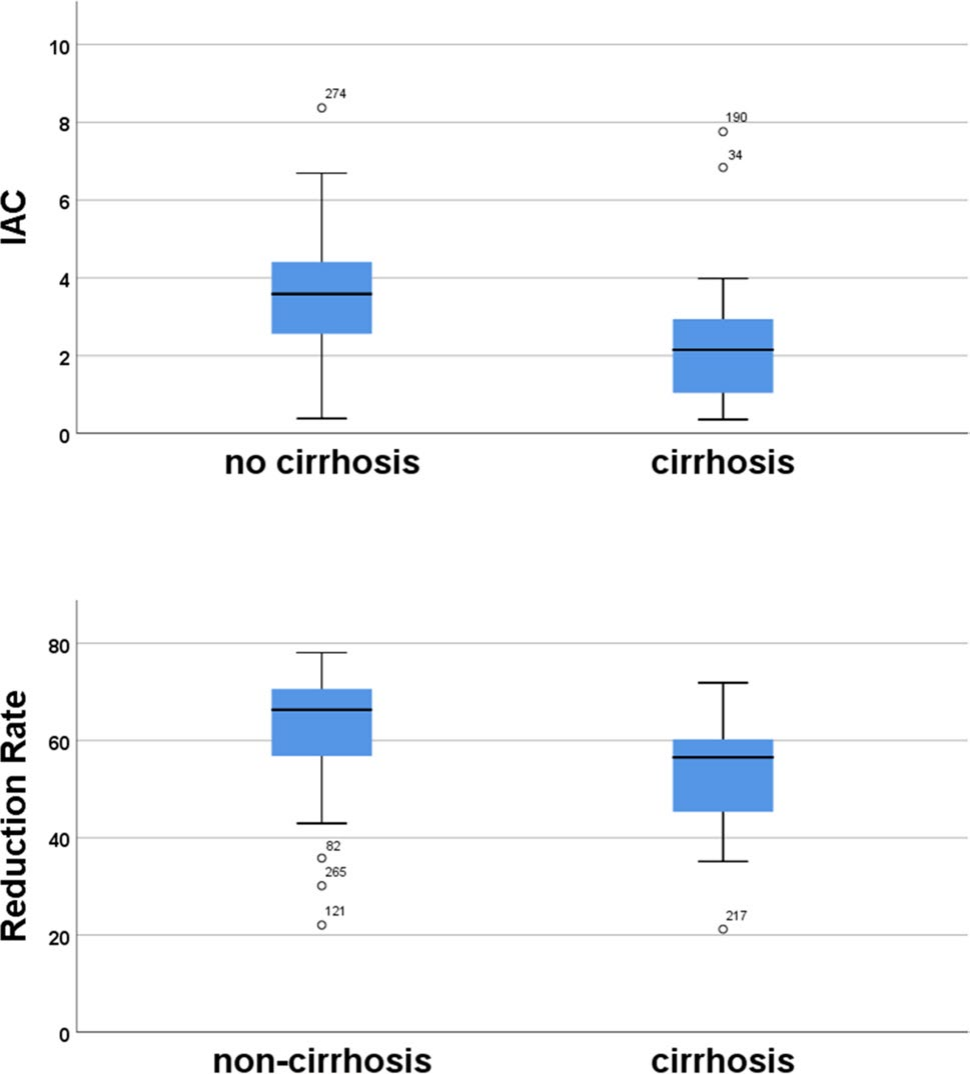
Boxplots depicting IAC and reduction rate values grouped into patients with no cirrhosis and those with cirrhosis.

Cirrhosis was present in 20 patients. IAC was 3.38 in non-cirrhotic organs and 2.50 in cirrhotic livers (p = 0.023). Reduction Rate was 65.6 in non-cirrhotic organs and 56.5 in cirrhotic livers (p = 0.002). Figure 3 shows the results as boxplots.

### Albumin

**Figure 4:**
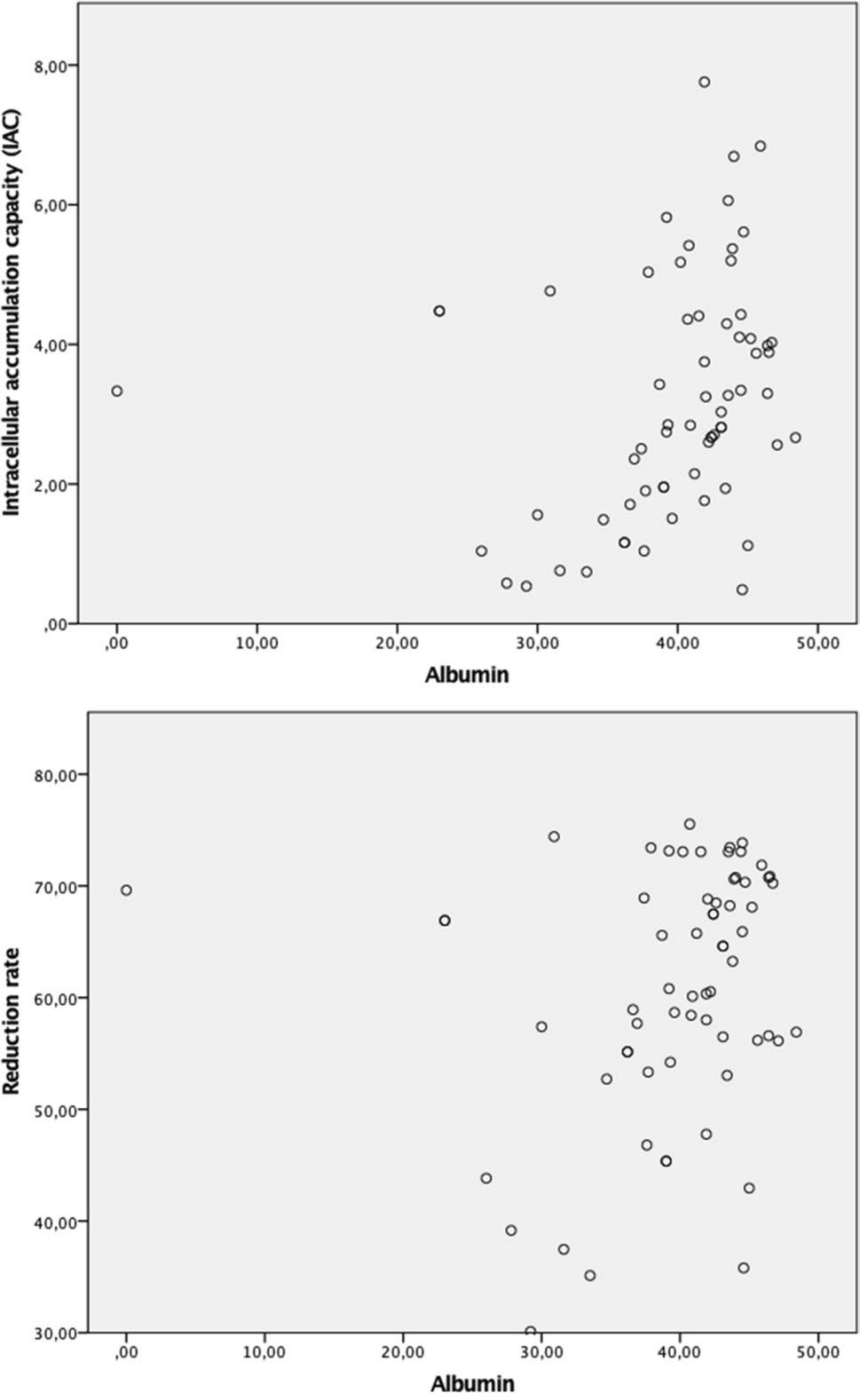
Scatterplot of albumin vs. IAC/RR.

As a simplified surrogate parameter for liver function, albumin was chosen and correlations with both RR and IAC were computed. Correlation, as expressed by Spearman’s ρ, was higher between IAC and albumin (ρ = 0.364, p = 0.003), than between RR and albumin (ρ = 0.285, p = 0.022) (Fig. 4), although both associations have to be classified as weak^15^.

## Discussion

Based on the findings of our exploratory investigation, we wish to propose the intracellular accumulation capacity (IAC) as a novel imaging marker for liver function on Gd-EOB-DTPA enhanced MR-imaging. IAC can be calculated easily from T1 mapping sequence image data, and it correlates slightly better with serum albumin as a simplified surrogate parameter of liver function than the reduction rate.

Our results show a close correlation between values of reduction rate and those of IAC. Equally, predictability of cirrhosis is comparable between both parameters. We thereby conclude, that the approach to calculate ECV using an extracellular contrast agent can be transferred to the intracellular space in hepatic imaging using the hepatocyte-specific contrast agent gadoxetate disodium.

Quantitative measurement of the myocardial tissue T1 time has long been used and is now well established for the assessment of diffuse myocardial fibrosis. Nevertheless, the accuracy of post contrast T1 mapping is sensitive to a lot of confounding factors, such as dose and concentration of the contrast agent, the time delay between contrast agent administration and image acquisition, the wash-out rate, and the hematocrit level, which affects the partition coefficient of the gadolinium contrast agent as gadolinium resides only in plasma and does not enter intact red blood cells^8,16^. Calibrating the T1 maps to blood including its hematocrit seemed to be a valuable tool to circumvent at least several of these confounding factors, and led to the description of a new parameter named ECV (extracellular volume)^9,10,17^. Calculation of the percentage of the noncellular tissue volume (ECV) is established in cardiac magnetic resonance imaging and is based on the assumption of an equilibrium between the intravascular, extracellular compartment and the extracellular compartment in organs such as the myocardium or the liver. The myocardial ECV is measured as the percent of tissue comprised of extracellular space, which is a physiologically intuitive unit of measurement and is independent of field strength^10^.

As most of the aforementioned confounding factors to myocardial T1 relaxation times also apply to hepatic relaxation times, we assumed, that description of liver function using gadoxetate disodium might also benefit from a calibration to blood including its hematocrit. Whereas extracellular contrast agents show an equilibrium between the extracellular space and the intravascular compartment without relevant intracellular uptake, with gadoxetate disodium, in the hepatobiliary phase, one may assume a mainly intracellular storage and a low-level equilibrium between intravascular und extracellular compartment^18^. Therefore, the adaptation of the ECV-formula on IAC includes a minor correction by subtracting the low-level amount of extracellular contrast agent. The thereby calculated IAC can range from close to 0, meaning no relevant intracellular accumulation with a gadoxetate retention similar to blood, to around eight, indicating a high intracellular accumulation capacity due to well-functioning hepatocytes, as Gd-EOB-DTPA uptake into hepatocytes depends on the integrity of the hepatic transport proteins^4,5,19,20^.

There are other approaches to quantify liver function using gadoxetate disodium of which most refer to signal intensity measurements and their derived measures liver-to-spleen ratio, liver-to-muscle-ratio, and relative enhancement^21–26^. Still, apart from field strength, signal intensities in MRI are also dependent on the manufacturer, the device, and also the sequence used, and, therefore, cannot be easily transferred, and in direct comparison, relaxation time measurements showed higher correlation to scintigraphically evaluated liver function than signal intensity-based measurements^21^.

Most recently, another approach using relaxometry was presented by Liu et al., using the hepatocyte enhancement fraction as a surrogate parameter for liver function, which is calculated by the T1 differences before and after the enhancement of liver and spleen using the double compartment model^27^. This approach appears to be promising, and a direct comparison to the approach presented here would be desirable. Still, it includes a comparison to another organ, the spleen, thereby possibly introducing new confounding factors.

To further evaluate the diagnostic value of IAC, we correlated it to serum albumin, which is one of the parameters that can be measured in peripheral blood and are used to assess liver synthetic function in clinical practice. Compared to reduction rate, the correlation was higher with IAC, a finding, which might support the assumption, that calibrating the T1 values to blood circumvents a few of the confounding factors limiting reproducibility of T1 values^10^ and thus might lead to more precise results. This might be of value at an earlier stage of reduced liver function, even though the test is not specific for liver disease: albumin serum levels are also reduced in patients with malnutrition or malabsorption, protein-losing enteropathy, or nephrotic syndrome, but, nevertheless, even though we cannot confidently exclude malnutrition as a confounding factor, according to the accessible medical records, the other typical preconditions for serum albumin alterations were not present in our study population.

Yet, in the present collective, prediction of cirrhosis was not improved by IAC compared to reduction rate. Due to the small number of patients with cirrhosis (n = 20) as well as the heterogeneity of its etiology as well as the disease’s stage, we regard these results as preliminary and cannot draw any final conclusion as to IAC’s value in cirrhotic livers.

### Limitations

There are some limitations to this investigation that need to be addressed: the study as approved by and registered with the local ethics committee did not include an additional blood sample for scientific reasons. Therefore, possibilities to measure liver function were limited as the only parameter which could be obtained in a relevant part of the patient collective was albumin, present in at least about 65% of patients. In a prospective approach, dedicated liver function tests such as the ICG or also the LIMAx test could have been applied, additionally, more patients with highly decreased liver function should be examined. Additionally, liver imaging was performed mainly for characterization of focal liver lesions—therefore the majority of patients included did not have relevantly impaired liver function. However, advantages of the IAC over the reduction rate are to be expected in patient populations with reduced liver function, therefore further studies are warranted to characterize the benefits of this imaging marker further.

## Conclusion

The formula to calculate ECV can be transferred to calculate the IAC of hepatocytes using the hepatocyte-specific contrast agent gadoxetate disodium. Additionally, IAC shows a higher correlation to albumin, thus possibly qualifying as a new image-based parameter to estimate synthetic liver function.
